# Evaluating the three-level approach of the U-smile method for imbalanced binary classification

**DOI:** 10.1371/journal.pone.0321661

**Published:** 2025-04-10

**Authors:** Barbara Więckowska, Katarzyna B. Kubiak, Przemysław Guzik

**Affiliations:** 1 Department of Computer Science and Statistics, Poznan University of Medical Sciences, Poznan, Poland; 2 Department of Cardiology - Intensive Therapy and Internal Medicine, Poznan University of Medical Sciences, Poznan, Poland; 3 University Centre for Sports and Medical Studies, Poznan University of Medical Sciences, Poznan, Poland; Khalifa University, UNITED ARAB EMIRATES

## Abstract

Real-life binary classification problems often involve imbalanced datasets, where the majority class outnumbers the minority class. We previously developed the U-smile method, which comprises the U-smile plot and the BA, RB and I coefficients, to assess the usefulness of a new variable added to a reference prediction model and validated it under class balance. In this study, we evaluated the U-smile method under class imbalance, proposed a three-level approach of the U-smile method, and used the I coefficients as a weighting factor for point size in the U-smile plots of the BA and RB coefficients. Using real data from the Heart Disease dataset and generated random variables, we built logistic regression models to assess four new variables added to the reference model (nested setting). These models were evaluated at seven pre-defined imbalance levels of 1%, 10%, 30%, 50%, 70%, 90% and 99% of the event class. The results of the U-smile method were compared to those of certain traditional measures: Brier skill score, net reclassification index, difference in F1-score, difference in Matthews correlation coefficient, difference in the area under the receiver operating characteristic curve of the new and reference models, and the likelihood-ratio test. The reference model overfitted to the majority class at higher imbalance levels. The BA-RB-I coefficients of the U-smile method identified informative variables across the entire imbalance range. At higher imbalance levels, the U-smile method indicated both prediction improvement in the minority class (positive BA and I coefficients) and reduction in overfitting to the majority class (negative RB coefficients). The U-smile method outperformed traditional evaluation measures across most of the imbalance range. It proved highly effective in variable selection for imbalanced binary classification, making it a useful tool for real-life problems, where imbalanced datasets are prevalent.

## Introduction

Prediction models for binary classification are important tools in various fields, including medicine, epidemiology, fraud detection, credit risk assessment, spam filtering and more, for classifying individuals or objects into two groups, called classes: the non-event or event class. Statistical and machine learning (ML) methods such as logistic regression (LR), artificial neural networks (ANN), K-nearest neighbours (KNN), support vector machines (SVM) and random forests (RF) are effective algorithms for binary classification [1–5]. Real-life binary classification problems often involve imbalanced datasets [6,7], where the distribution of classes is skewed and the majority class outnumbers the minority class.

Imbalanced classification still poses many challenges for model training and evaluation [6–11]. Firstly, models trained on imbalanced datasets may overfit to the majority class and underfit the minority class. This leads to poor performance in the minority class, especially on unseen data. As some ML algorithms may be inherently biased to the majority class and be more sensitive to class imbalance than others, various strategies have been proposed to address this issue [12–15]. Secondly, many traditional evaluation metrics, such as accuracy or Brier score (BS), prioritise the majority class, while others, such as F1-score or the precision-recall (PR) curve, prioritise the event class, which is often the minority class. Moreover, these measures do not provide separate scores for each class, i.e., they are not stratified by class. Although one class (usually the minority class) is often of greater interest in imbalanced classification, making a separate evaluation in each class is particularly important. Unlike assessing the overall model performance with traditional performance measures, evaluating each class separately can lead to more informed decisions regarding model construction. As a result, many traditional metrics can be inappropriate or even misleading for imbalanced classification. Therefore, the performance of prediction models for imbalanced binary classification depends on using accurate, interpretable and reliable evaluation measures and variable selection strategies.

**Fig 1:**
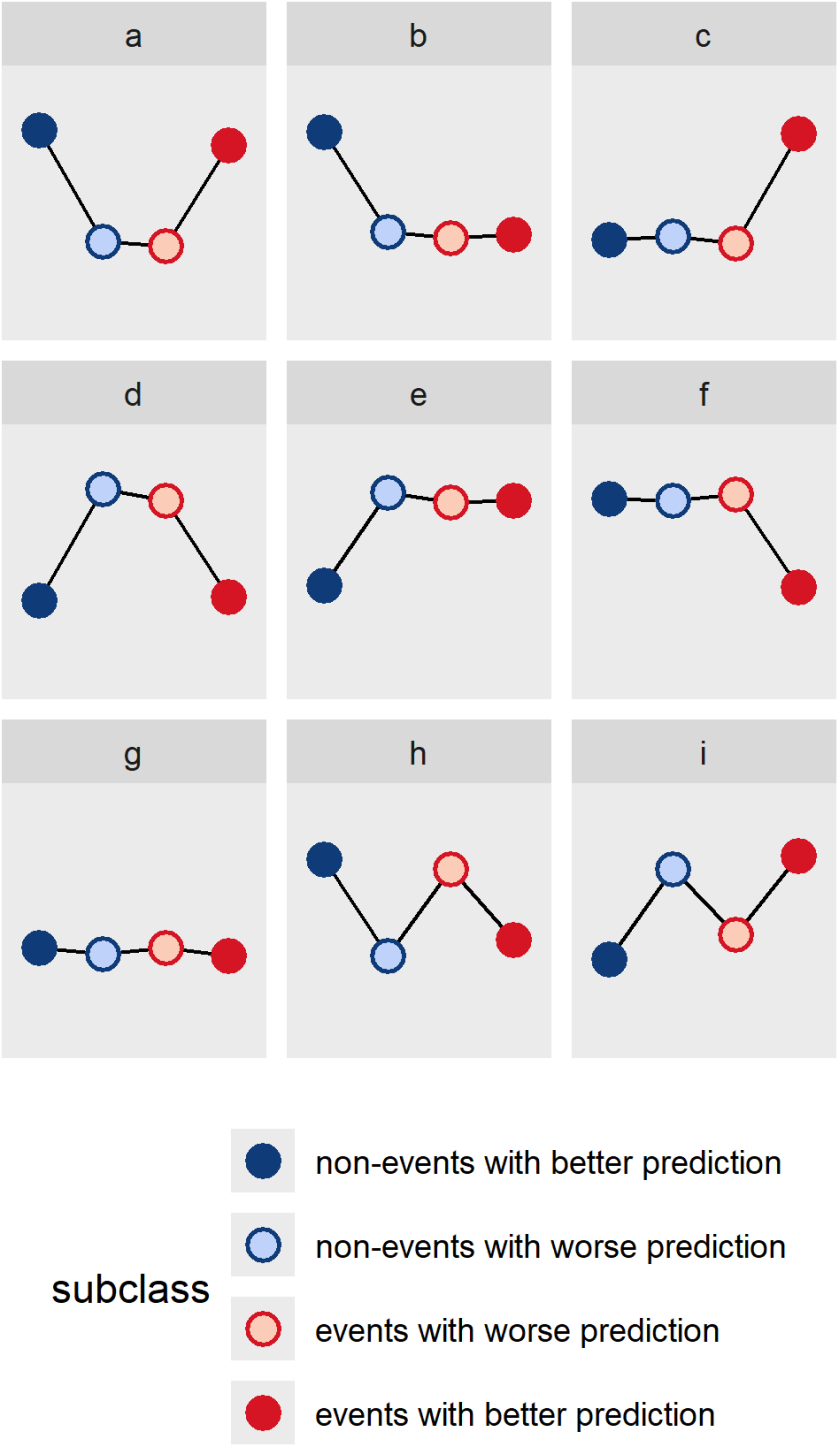
Some possible shapes of the U-smile plot. The U-smile plot of the BA or RB coefficients represents the effect of adding a new variable to a reference model. Its shape can indicate improved prediction for both classes (double smile – panel a) or either class with no changes in the other (smile + flat line, flat line + smile – panels b and c). It can also show worsened prediction for both classes (double frown - panel d) or either class with no changes in the other (frown + flat line, flat line + frown – panels e and f). A flat line of the entire U-smile plot indicates no change in prediction for both classes (double flat line – panel g), while a zigzag pattern shows improved prediction for one class and worsened prediction for the other (smile + frown, frown + smile – panels h and i).

The U-smile method [16], previously developed by our team, evaluates the effect of adding a new variable to a reference prediction model by comparing the new and reference models. Through stratification by class, it provides separate assessment for non-events and events. Our previous work introduced a two-level approach of the U-smile method. At level 1, each class is further split into two subclasses: one with better and the other with worse prediction. The average absolute and relative changes in prediction are quantified with the BA and RB coefficients, respectively, and visualised on their U-smile (BA) and U-smile (RB) plots. Visual assessment of the U-smile plot’s shape allows for an immediate and intuitive interpretation of whether adding a new variable to the reference model improved (a smile), worsened (a frown) or did not affect (a flat line) the reference prediction separately for each class. Fig 1 shows some possible shapes of the U-smile plot. Then, at level 2, the net BA and RB coefficients are determined as differences between improvement and worsening coefficients to quantify net changes in each class. The U-smile method enables to identify the most informative variables in each class, given a set of reference variables, and provides more insight into variable selection that the likelihood-ratio test (LRT) or the difference in the area under the receiver operating characteristic (ROC) curve (ΔAUC) of the new and reference models.

So far, we have evaluated the U-smile method for nested models differing by one variable, using both real and artificial data, under both informative and non-informative scenarios with nearly balanced data. Namely, the percentage of the event class was 47.4% and 47.6% in the training and test datasets, respectively. We compared the results of the U-smile method with those of the LRT and ΔAUC of ROC curves. However, we have not yet investigated the performance and applicability of the U-smile method under class imbalance.

In this methodological study, we aim (1) to evaluate the U-smile method for binary classification under class imbalance ranging from 1% to 99% of the event class, (2) to propose a new overall level 3 with weighted overall BA, RB and I coefficients, creating a three-level approach of the U-smile method, and (3) to introduce a novel approach of using the I coefficient as a weighting factor for point size in the U-smile (BA) and U-smile (RB) plots. We also aim to compare the U-smile method to certain traditional evaluation measures for model comparison: the LRT, Brier skill score (BSS) [17,18], net reclassification index (NRI) [19], difference in F1-score (ΔF1-score) [20], difference in Matthews correlation coefficient (ΔMCC) [21] and ΔAUC under ROC curves [22] of the new and reference models.

## Materials and methods

### The U-smile method

The U-smile method evaluates the usefulness of a new variable added to a reference model by comparing the new model with the reference model. It consists of the U-smile plot and the BA-RB-I coefficients, offering both graphical and numerical assessment (quantitative measures, i.e., the BA and RB coefficients, and counting measures, i.e., the I coefficients). The U-smile method is based on comparing the predicted probabilities of the new and reference models, and their residuals, δ, i.e., the differences between the true outcome and the predicted probability. Equivalently, model residuals are distances on the probability axis between these two values and are considered prediction errors.

We denote the non-event and event classes with subscripts _0_ and _1_, respectively, and prediction improvement and worsening are denoted with superscripts ^+^ and ^–^, respectively. Additionally, subscript _(ref)_ denotes the reference model, and no subscript denotes the new model. This article provides a conceptual (and interpretative) overview of the U-smile method, for mathematical details and formulas, please refer to our previous work [16].

#### The prediction improvement-worsening (PIW) matrix.

**Fig 2:**
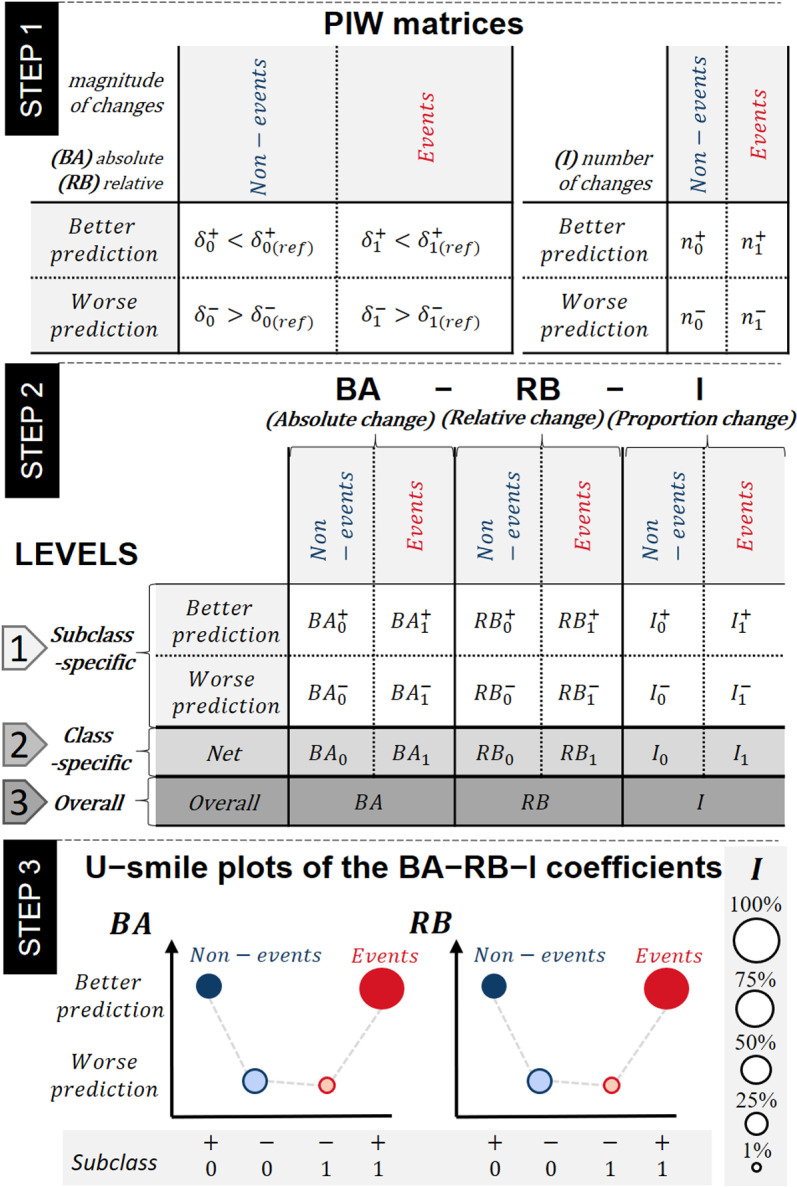
A step-by-step guide to constructing the BA-RB-I coefficients and the U-smile plot. Subscripts _0_ and _1_ denote the non-event and event classes, respectively, superscripts ^+^ and ^–^ denote better and worse prediction of the new model as compared to the reference model, respectively, δ_(ref)_ and δ are the residuals of the reference and new models, respectively, and n is the number of individuals in the group indicated by sub- and superscripts. BA coefficients: average absolute changes in prediction between new and reference models; RB coefficients: relative changes in prediction between new and reference models (relative to the prediction error of the reference model); I coefficients: proportions of the prediction changes in each class. **Step 1.** Prediction improvement-worsening (PIW) matrices present the four subclasses resulting from cross-tabulating prediction changes with the true outcome. Better prediction means that the new residuals were smaller than the reference ones, worse prediction means that they were larger than the reference ones. The left PIW matrix shows comparisons of residuals of the new and reference models. The right PIW matrix shows the number of individuals in each subclass. **Step 2.** The three-level approach of the U-smile method. At level 1, in each of the four subclasses, the magnitude of changes in the predicted probabilities (expressed by model residuals) is transformed into BA and RB coefficients, and the number of individuals is transformed into the I coefficients. At level 2, the net coefficients are determined for each class as differences of the subclass-specific coefficients of level 1, i.e., improvement coefficient less worsening coefficient. At level 3, the weighted overall BA-RB-I coefficients are calculated as weighted means of their respective net coefficients. **Step 3.** The U-smile plot of the BA-RB-I coefficients. The four subclasses are plotted on the x-axis in a specific order, and the values of the BA and RB coefficients are plotted on the y-axis. The point size is scaled according to the value of the respective I coefficient. The point colour and fill indicate the class and subclass, respectively: points of non-events are blue, points of events are red, points of subclasses with better prediction are solid-filled, and points of subclasses with worse prediction are lighter-filled.

The starting point of the U-smile method (Fig 2 Step 1) is cross-tabulating prediction changes with the true outcome to identify four subclasses of individuals, *i*:

- $i_{0}^{+}$, non-events with better prediction,
- $i_{0}^{-}$, non-events with worse prediction,
- $i_{1}^{-}$, events with worse prediction,
- $i_{1}^{+}$, events with better prediction.

The prediction improvement-worsening matrix (PIW) is based on model residuals. Better predictions occur when residuals of the new model (δ) are smaller than the reference model’s residuals (δ_(ref)_), while worse predictions correspond to larger residuals. Additionally, the PIW matrix provides cardinalities of each subclass, i.e., the numbers of individuals, n, in each subclass: $n_{0}^{+}$, $n_{0}^{-}$, $n_{1}^{-}$, $n_{1}^{+}$, respectively. This concept is analogous to the commonly used confusion matrix that cross-tabulates the actual class with the model’s predicted class.

#### Three levels of the BA-RB-I coefficients.

The BA (absolute change), RB (relative change), and I (proportion) coefficients quantify prediction changes and are calculated at three levels (Fig 2 Step 2).

At level 1, the twelve subclass-specific coefficients measure prediction changes for all four subclasses, as defined in our previous work [16]: $BA_{0}^{+}$, $BA_{0}^{-}$, $BA_{1}^{-}$, $BA_{1}^{+}$, $RB_{0}^{+}$, $RB_{0}^{-}$, $RB_{1}^{-}$, $RB_{1}^{+}$ and $I_{0}^{+}$, $I_{0}^{-}$, $I_{1}^{-}$, $I_{1}^{+}$. We also defined six net coefficients of level 2 to summarise prediction changes in each class:


$$BA_{0}=BA_{0}^{+}-BA_{0}^{-},$$

$$BA_{1}=BA_{1}^{+}-BA_{1}^{-},$$

$$RB_{0}=RB_{0}^{+}-RB_{0}^{-},$$

$$RB_{1}=RB_{1}^{+}-RB_{1}^{-},$$

$$I_{0}=I_{0}^{+}-I_{0}^{-},$$

$$I_{1}=I_{1}^{+}-I_{1}^{-}.$$

The net I coefficients were previously reported as NRI_0_ and NRI_1_ [19].

In this study, we propose level 3 of the BA-RB-I coefficients. The weighted overall BA-RB-I coefficients summarise the general impact of the new variable and are defined as follows:


$$BA=w_{0}BA_{0}+w_{1}BA_{1},BA\epsilon \left[-1,1\right],$$

$$RB=w_{0}RB_{0}+w_{1}RB_{1},RB\epsilon \left(-\infty ,1\right],$$

$$I=w_{0}I_{0}+w_{1}I_{1},I\epsilon \left[-1,1\right],$$

where w_0_ = n_0_/n, and w_1_ = n_1_/n are weights for non-event and event class, respectively.

This approach is justified for imbalanced datasets at it ensures a proportional representation of both classes, reduces bias and reflects real-world scenarios. In the case of perfect class balance, the weights are equal and the weighted mean simplifies to the arithmetic mean. Using weighting improves the interpretation of the overall coefficients.

As a result of using cardinality-based weights, the weighted overall BA coefficient is equivalent to ΔBS, the weighted overall RB coefficient is connected to BSS (we obtain BSS using residual-based weights instead) [16], and the weighted overall I coefficient is the weighted version of the unweighted NRI.

The four subclass-specific coefficients are always positive. The class-specific net coefficients and the weighted overall coefficients are positive if the new model’s predictions are better than those of the reference model, and negative if they are worse.

#### The U-smile plot.

The U-smile plot (Fig 2 Step 3) visualises the subclass-specific coefficients by plotting the four subclasses in a fixed order, i.e. $i_{0}^{+}$, $i_{0}^{-}$, $i_{1}^{-}$, $i_{1}^{+}$, on the x-axis, the BA and RB values on the y-axis, and using the I coefficients for point size. In this way, the U-smile (BA) and U-smile (RB) plots are obtained. Points of the non-event and event classes are in blue and red, respectively, solid-filled points indicate better prediction, and lighter-filled points indicate worse prediction.

Shapes of the U-smile plot (Fig 1) – smiles, frowns, flat lines or zigzags – reflect prediction changes between new and reference models, with vertical distances showing the net coefficients. Larger changes yield high smiles or frowns, indicating significant model improvement or deterioration.

Instead of plotting separate U-smile plots of the I coefficients, we introduce a novel approach in this study: using the I coefficient as a weighting factor for point size in the U-smile (BA) and U-smile (RB) plots. This is in accordance with a recommendation from our previous work, where we advocated for the joint interpretation of the complementary BA-RB and I coefficients [16]. As a result, additional information about the proportion of individuals with prediction changes in each class was incorporated into the U-smile plot, ensuring a comprehensive assessment of a new variable.

### Data and models

We used the same data as in our previous work [16], i.e., the Heart Disease dataset [23] (available from the Machine Learning Repository [24], last accessed December 13, 2022), and generated random variables from probability distributions. A detailed description of preparing the Heart Disease dataset and generation of random variables is provided in our previous work. The prepared raw dataset is available on GitHub at https://github.com/kbkubiak/U-smile/tree/main/data.

The predicted outcome variable, $D,D\in \left\{0,1\right\},$ is the presence of coronary artery disease (confirmed by coronary angiography and defined as >50% diameter narrowing in any major vessel) in a patient (1 = having the disease/event, 0 = healthy/non-event). Building on our previous report, we used the same set of reference variables, X, from the Heart Disease dataset to build the reference model:

- Age in years,
- Sex (1 = male, 0 = female),
- Resting systolic blood pressure in mmHg on hospital admission (SBP),
- Serum cholesterol level in mg/dl (Chol).

As we have shown, the U-smile method reliably distinguishes between informative and non-informative candidate variables added to the reference model, regardless of whether they are numerical or categorical, or their distribution. Adding an informative variable improves the reference model’s performance, while adding a non-informative variable does not improve the model’s performance and might add noise. Therefore, from the eighteen (six real from the Heart Disease dataset and twelve random) previously tested candidate variables, we included only four in the current analysis, two real and two random ones:

- $Y_{1}$, ST depression (real, numerical, informative): exercise-induced ST depression relative to rest in mm,
- $Y_{2}$, glucose (real, categorical, non-informative): fasting blood glucose concentration > 120 mg/dl (1 = yes, 0 = no),
- $Y_{3}$, Str Rnd normal (random, numerical, informative): a random variable with normal distribution stratified by class - $N\left(10,2\right)$ for the non-event class and $N\left(12,2\right)$ for the event class,
- $Y_{4}$, Rnd normal (random, numerical, non-informative): a random variable with a standard normal distribution $N\left(0,1\right)$.

Following our previous work, the reference model was build using logistic regression and is given by:


$$logitP\left(D=1\text{|}X\right)=\alpha_{0}+\alpha_{1}Age+\alpha_{2}Sex+\alpha_{3}SBP+\alpha_{4}Chol.$$

The four new models, each built by adding one candidate variable $Y_{j},j=1,\ldots ,4,$ to the reference model (nested setting), are hence given by:


$$logitP\left(D=1\text{|}X,Y_{j}\right)=\alpha_{0}+\alpha_{1}Age+\alpha_{2}Sex+\alpha_{3}SBP+\alpha_{4}Chol+\alpha_{5}Y_{j}.$$

The raw dataset comprised 661 observations, including 347 non-events and 314 events. We pre-selected seven imbalance levels of 1%, 10%, 30%, 50%, 70%, 90% and 99% of the event class. To account for dataset variability, for each imbalance level, 1000 training datasets (300 observations) were sampled with replacement from the raw dataset, and corresponding 1000 test datasets (100 observations) were sampled with replacement from the remainder of the raw dataset. Models were fitted on each training dataset and validated on its corresponding test dataset.

In this work, the term “imbalance” means the percentage of the event class in the dataset. The following classification is used for imbalance levels: 1% and 99% - extreme imbalance, 10% and 90% - high imbalance, 30% and 70% - intermediate imbalance, 50% - (perfect) balance.

### Evaluation of the reference model and new variables

We evaluated the reference model’s performance at pre-selected imbalance levels of 1%, 10%, 30%, 50%, 70%, 90% and 99% of the event class. For each imbalance level, we calculated error measures, i.e., BS [17], stratified BS for non-events (BS_0_) and stratified BS for events (BS_1_) [25], and performance measures, i.e., AUC, F1-score and MCC, of 1000 reference models derived from the training and test datasets. Results were averaged across 1000 iterations. To assess overfitting to the majority class, we used BS_0_ and BS_1_, and their difference between test and training datasets.

To evaluate the four candidate variables, we compared the reference and new models across class imbalance ranging from 1% to 99% using multiple measures and methods listed below.

1. The U-smile method and the three levels of the BA-RB-I coefficients as described above.
2. Each new model was compared with the reference model using the LRT to assess statistical significance of each new variable. P-values were determined based on the mean value of the test statistic across 1000 iterations.
3. BSS [17,18] and NRI [19] are overall measures, whose level of generalizability corresponds to level 3 of the BA-RB-I coefficients. Their formulas are as follows:

$$BSS=1-\frac{BS}{BS_{ref}},BSS\epsilon \left(-\infty ,1\right],$$

where BS_ref_ and BS are Brier scores of the reference and new models, respectively, and express mean squared (prediction) error of each model,


$$NRI=NRI_{0}+NRI_{1},NRI\epsilon \left[-2,2\right],$$

where NRI_0_ = I_0_ and NRI_1_ = I_1_. We decided to use BSS and NRI as they are connected to the overall RB and I and differ only by the weights used.

1. The difference in AUC of ROC curves of the new and reference models, ΔAUC, $\Delta AUC=AUC–AUC_{ref}$, was assessed using DeLong’s test for two correlated ROC curves [26]. P-values were determined based on the mean value of the test statistic across 1000 iterations.
2. The differences in F1-score, ΔF1-score [20], and in MCC, ΔMCC [21], of the new and reference models. F1-score and MCC are calculated using a 2x2 confusion matrix (Table 1) that shows cross-tabulation of the actual class with the model’s predicted class (based on the conventional probability threshold of 0.5 [27,28]). F1-score is calculated using TP, FP and FN, but not TN. By contrast, MCC is calculated using all four values from the confusion matrix. The formulas for determining F1-score, MCC, ΔF1-score and ΔMCC are as follows:

**Table 1:** The confusion matrix shows a cross-tabulation of the actual class with the model’s predicted class (based on the conventional probability threshold of 0.5).

		Predicted class	
		Event	Non-event
**Actual class**	**Event**	TP	FN
	**Non-event**	FP	TN

TP, number of true positives; FN, number of false negatives; TN, number of true negatives; FP, number of false positives.


$$F1-score=\frac{2TP}{2TP+FP+FN},$$

$$MCC=\frac{\left(TP\times TN\right)-\left(FP\times FN\right)}{\sqrt{\left(TP+FP\right)\left(TP+FN\right)\left(TN+FP\right)\left(TN+FN\right)}},$$

$$\Delta F1-score=F1-score–F1-score_{ref},\Delta F1-score\epsilon \left[-1,1\right],$$

$$\Delta MCC=MCC–MCC_{ref},\Delta MCC\epsilon \left[-2,2\right].$$

Subscript _ref_ indicates the reference model and no subscript indicates a new model. Positive values of BSS, NRI, ΔAUC, ΔF1-score and ΔMCC mean improved performance of the reference model, negative values mean performance worsening, and zero means no change in performance upon adding the new variable.

To visualise coefficient trends in subclasses (level 1), classes (level 2) and overall data (level 3), we plotted the BA-RB-I coefficients against the percentage of the event class and fitted smooth curves using the local polynomial regression fitting (LOESS) method.

For the LRT and DeLong’s test for two correlated ROC curves, the conventional significance level of α = 0.05 was adopted. Analyses were performed using R Statistical Software (version 4.4.1; R Core Team, 2024) [29] and RStudio (version 2024.4.2.764; Posit Software, PBC, 2024) [30].

## Results

### Evaluation of the reference model

Due to the planned expansion of the reference model, a detailed analysis of its current performance was first conducted. Table 2 shows values of evaluation measures for the reference model derived from the training and test datasets across imbalance ranging from 1% to 99% of the event class. The error measures (BS, BS_0_ and BS_1_) were higher on the test dataset than on the training dataset, while the performance measures (AUC, F1-score and MCC) were higher on the training dataset than on the test dataset. In particular, we observe that F1-score increased across the entire imbalance range.

**Table 2:** Values of the evaluation measures for the reference model derived from the training and test datasets across imbalance ranging from 1% to 99% of the event class.

	Training dataset Percentage of the event class							Test dataset Percentage of the event class						
	1%	10%	30%	50%	70%	90%	99%	1%	10%	30%	50%	70%	90%	99%
**AUC**	0.854	0.762	0.755	0.752	0.754	0.762	0.849	0.674	0.728	0.737	0.738	0.735	0.735	0.633
**BS**	0.009	0.081	0.174	0.201	0.171	0.078	0.009	0.011	0.086	0.181	0.209	0.179	0.083	0.011
**BS**_**0**_	0.001	0.015	0.090	0.207	0.374	0.648	0.845	0.002	0.017	0.094	0.214	0.388	0.673	0.938
**BS**_**1**_	0.880	0.675	0.370	0.196	0.084	0.015	0.001	0.954	0.702	0.384	0.203	0.089	0.017	0.002
**F1**	0.039	0.055	0.438	0.692	0.835	0.950	0.995	0.003	0.032	0.414	0.682	0.826	0.948	0.994
**MCC**	0.514 (913)	0.127 (371)	0.319	0.363	0.330	0.240 (89)	0.471 (845)	0.029 (912)	0.086 (638)	0.292	0.344	0.298	0.245 (334)	0.074 (864)

AUC, area under the receiver operating characteristic curve; BS, Brier score; BS_0_, stratified Brier score for non-events; BS_1_, stratified Brier score for events; F1, F1-score; MCC, Matthews correlation coefficient.

Shown are mean values from 1000 iterations. The number of iterations for which MCC could not be calculated is shown in parentheses.

**Fig 3:**
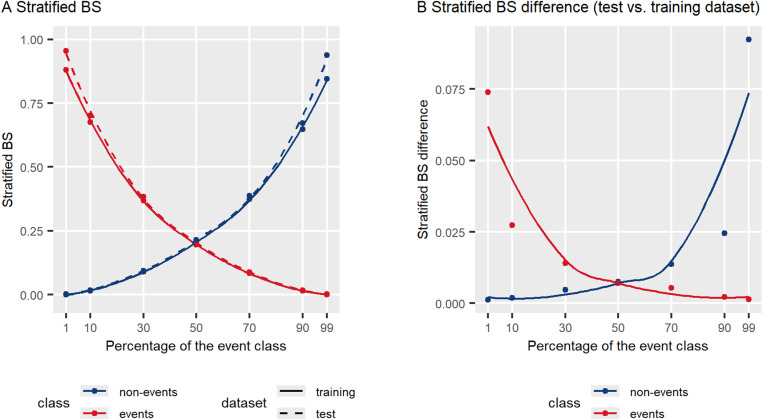
Prediction error of the reference model in each class, quantified by the stratified Brier score (BS), across imbalance ranging from 1% to 99% of the event class. Panel A shows results for the training and test datasets, while panel B shows the differences in stratified BS between test and training datasets. Points represent mean values from 1000 iterations. Smooth curves were fitted using the local polynomial regression fitting (LOESS) method.

Fig 3 shows the prediction error of the reference model in each class, expressed as the stratified BS, against the percentage of the event class in the dataset. We observe overfitting to the majority class at high and extreme imbalance levels. Namely, as the percentage of a class in the dataset increased, its prediction error decreased, approaching zero at high and extreme imbalance levels (panel A). Panel B shows the differences in stratified BS between test and training datasets, i.e., the distance between curves representing both datasets in panel A. The largest differences, meaning the poorest performance on unseen data, occurred in the minority class at high and extreme imbalance levels. Also, there was a slight asymmetry between the classes (panel A): even at perfect class balance (50% events and 50% non-events), the model showed a slight bias towards predicting the event class as BS_1_ was lower than BS_0_.

### Level 1: U-smile plots of the subclass-specific BA-RB-I coefficients

**Fig 4:**
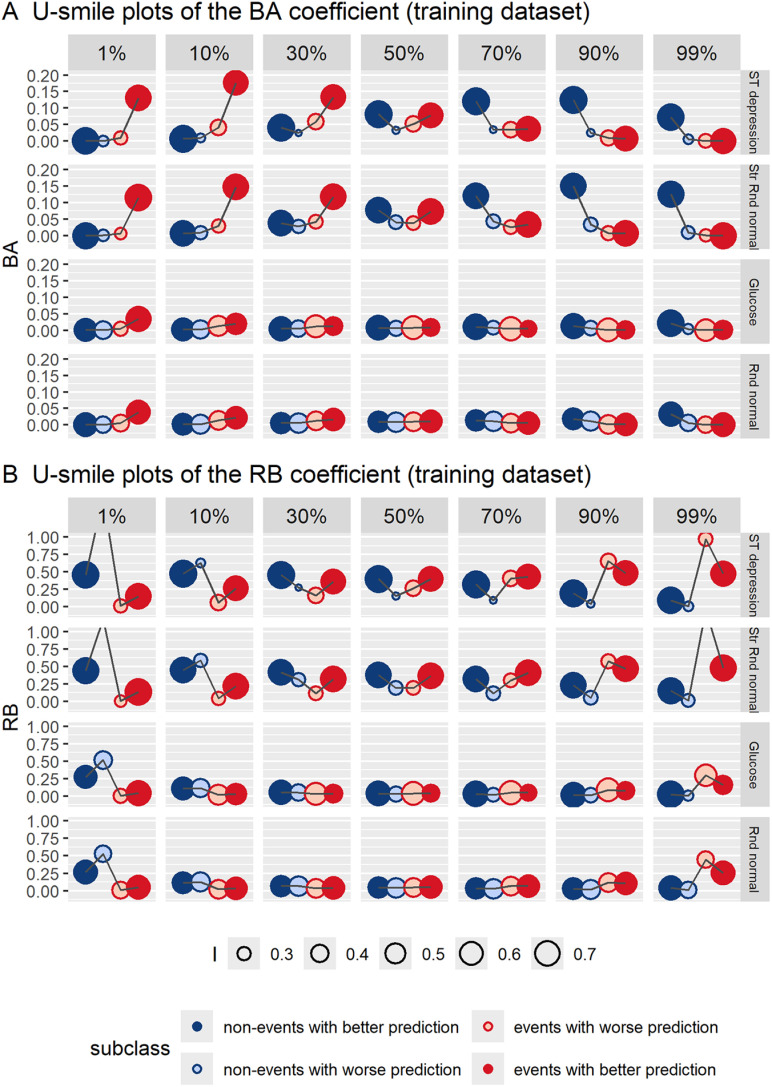
U-smile plots of the subclass-specific BA-RB-I coefficients across imbalance ranging from 1% to 99% of the event class, for four new models derived from the training dataset. Two informative variables (ST depression and Str Rnd normal) and two non-informative variables (glucose and Rnd normal) were added to the reference model. The plotted coefficient values represent means from 1000 iterations. The I coefficient is the weighting factor for point size. BA coefficients: average absolute changes in prediction between new and reference models; RB coefficients: relative changes in prediction between new and reference models (relative to the reference prediction error); I coefficients: proportions of individuals with prediction changes in each class.

**Fig 5:**
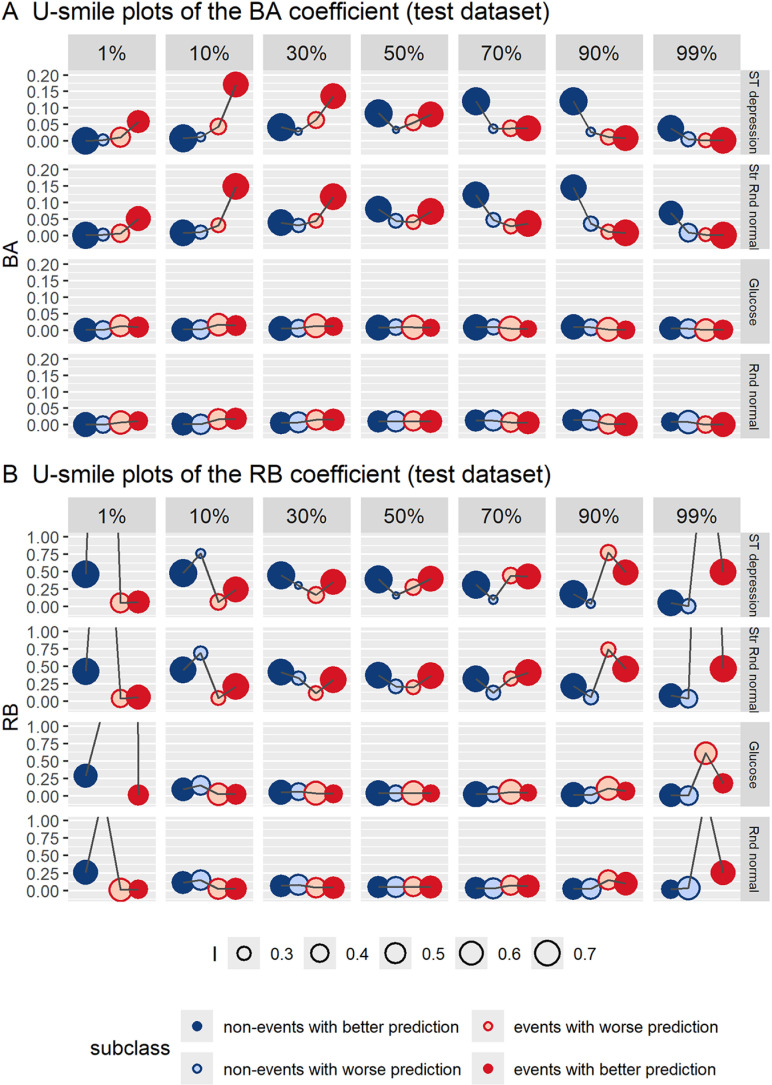
U-smile plots of the subclass-specific BA-RB-I coefficients across imbalance ranging from 1% to 99% of the event class, for four new models derived from the test dataset. Two informative variables (ST depression and Str Rnd normal) and two non-informative variables (glucose and Rnd normal) were added to the reference model. The plotted coefficient values represent means from 1000 iterations. The I coefficient is the weighting factor for point size. BA coefficients: average absolute changes in prediction between new and reference models; RB coefficients: relative changes in prediction between new and reference models (relative to the reference prediction error); I coefficients: proportions of individuals with prediction changes in each class.

Figs 4 and 5 show U-smile plots of the subclass-specific BA-RB-I coefficients for new models derived from the training and test datasets, respectively, across imbalance ranging from 1% to 99%. S1 Table shows the values of the subclass-specific BA-RB-I coefficients for both training and test datasets (mean values from 1000 iterations).

For informative variables, i.e., ST depression and Str Rnd normal, both U-smile (BA) and U-smile (RB) plots showed a double, approximately symmetrical, smile shape at 50%. At higher imbalance levels the U-smile (BA) plots had higher smiles for the minority class than at balance, and became flat for the majority class. With increasing imbalance level, smiles of the RB coefficient persisted for the minority class, while for the majority class, they lowered and turned to frowns at high and extreme imbalance levels. Frowns of the RB coefficients mean that the prediction error of the new model was larger than that of the reference model. The point size, representing the value of the I coefficient, was larger for subclasses with better prediction than for those with worse prediction.

Across the entire imbalance range, the U-smile (BA) and U-smile (RB) plots showed concordant smile shapes for the minority class. With increasing imbalance level, the shapes of the U-smile (BA) and U-smile (RB) plots for the majority class increasingly diverged. The discrepancy was most pronounced at high and extreme imbalance levels: the U-smile (BA) plots were flat, while the U-smile (RB) plots produced frowns, indicating no visible average prediction change but worsened model prediction for this class as compared to the reference model.

For non-informative variables, i.e., glucose and Rnd normal, both U-smile (BA) and U-smile (RB) plots remained flat across almost the entire range of imbalance, except for extreme imbalance levels, where we observe low smiles for the minority class in the U-smile (BA) plots, and low frowns for the majority class in the U-smile (RB) plots.

Regarding the results obtained from the test dataset (Fig 5), the U-smile (RB) plots had the same or very similar shapes for both the training and test datasets across all levels of imbalance. Similarly, the U-smile (BA) plots were consistent between the training and test datasets, except for the informative variables at extreme imbalance levels, where they showed smaller prediction improvement in the minority class compared to the training dataset. The I coefficients were less reproducible between the training and test datasets compared to the high reproducibility observed for the BA and RB coefficients. Despite these discrepancies, the results led to comparable interpretations and conclusions.

### Level 2: The class-specific net BA-RB-I coefficients

Tables 3 and S2 show the values of the class-specific net BA-RB-I coefficients for models derived from the training and test datasets, respectively, across imbalance ranging from 1% to 99%. Negative values of the net coefficients indicate prediction worsening for a given class. We observe the largest number of negative values in the net RB coefficient for the majority class at high and extreme imbalance levels. Although there were also some individual negative values (almost zero) in the net BA coefficients for the minority class, we considered them to be zero. At high and extreme imbalance levels, the net BA and RB were concordant in sign for the minority class, i.e., both were positive, while the net BA was zero and the net RB was negative for the majority class. This was also visible in the U-smile (BA) and U-smile (RB) plots.

**Table 3:** Level 2: Values of the class-specific net BA-RB-I coefficients for models derived from the training dataset across imbalance ranging from 1% to 99% of the event class.

New model	Level 2 coefficient	Training dataset Percentage of the event class						
		1%	10%	30%	50%	70%	90%	99%
**Reference model + ST depression**	**BA**_**0**_	**0.000**	**-0.002**	0.016	0.050	0.087	0.100	0.066
	**BA**_**1**_	0.121	0.136	0.074	0.026	0.002	**-0.002**	**0.000**
	**RB**_**0**_	**-1.122**	**-0.149**	0.179	0.240	0.231	0.155	0.082
	**RB**_**1**_	0.138	0.201	0.201	0.133	0.026	**-0.171**	**-0.495**
	**I**_**0**_	0.481	0.528	0.550	0.547	0.549	0.544	0.511
	**I**_**1**_	0.396	0.296	0.303	0.301	0.311	0.318	0.372
**Reference model + Str Rnd normal**	**BA**_**0**_	**0.000**	**-0.002**	0.009	0.038	0.077	0.117	0.117
	**BA**_**1**_	0.109	0.118	0.075	0.035	0.009	**-0.001**	**0.000**
	**RB**_**0**_	**-0.739**	**-0.144**	0.104	0.182	0.206	0.180	0.139
	**RB**_**1**_	0.125	0.175	0.202	0.176	0.104	**-0.106**	**-0.831**
	**I**_**0**_	0.454	0.398	0.398	0.395	0.388	0.380	0.426
	**I**_**1**_	0.472	0.396	0.385	0.381	0.376	0.375	0.430
**Reference model + Glucose**	**BA**_**0**_	**0.000**	**0.000**	0.000	0.001	0.003	0.007	0.017
	**BA**_**1**_	0.030	0.007	0.002	0.001	0.000	**0.000**	**0.000**
	**RB**_**0**_	**-0.243**	**-0.011**	0.004	0.007	0.009	0.011	0.022
	**RB**_**1**_	0.036	0.010	0.006	0.004	0.001	**-0.013**	**-0.139**
	**I**_**0**_	0.159	0.130	0.254	0.327	0.380	0.348	0.504
	**I**_**1**_	0.365	0.020	**-0.157**	**-0.236**	**-0.274**	**-0.208**	**-0.115**
**Reference model + Rnd normal**	**BA**_**0**_	**0.000**	**0.000**	0.000	0.002	0.003	0.007	0.026
	**BA**_**1**_	0.032	0.009	0.003	0.001	0.000	**0.000**	**0.000**
	**RB**_**0**_	**-0.263**	**-0.014**	0.005	0.007	0.009	0.011	0.033
	**RB**_**1**_	0.038	0.014	0.009	0.006	0.003	**-0.012**	**-0.192**
	**I**_**0**_	0.239	0.065	0.042	0.038	0.043	0.065	0.242
	**I**_**1**_	0.236	0.110	0.098	0.093	0.096	0.099	0.240

BA coefficients: average absolute changes in prediction between new and reference models; RB coefficients: relative changes in prediction between new and reference models (relative to the reference prediction error); I coefficients: proportions of individuals with prediction changes in each class.

Shown are mean values from 1000 iterations. Negative values are highlighted in bold type.

Across most of the imbalance range, both net BA and net RB coefficients were higher for informative than for non-informative variables. At high and extreme imbalance levels, the net BA_0_ and BA_1_ were approximately zero for both informative and non-informative variables when their respective class was the majority class. At these levels, the net RB_0_ and RB_1_ were negative and lower for informative than for non-informative variables when their respective class was the majority class. For non-informative variables, values of the class-specific net BA and RB coefficients remained relatively constant and around zero across the entire imbalance range. The net I coefficients were lower for non-informative variables than for informative variables across the entire imbalance range, except for higher I_0_ for glucose (a non-informative variable) than for Str Rnd normal (an informative variable) when the imbalance exceeded approximately 90%.

### Level 3: The weighted overall BA-RB-I coefficients and traditional performance measures

Tables 4 and S3 show the values of the weighted overall BA-RB-I coefficients and traditional performance measures for models derived from the training and test datasets, respectively, across imbalance ranging from 1% to 99%. S1 and S2 Figs show the ROC curves of the new and reference models derived from the training and test datasets, respectively, across this range of class imbalance.

**Table 4:** Level 3: Values of the weighted overall BA-RB-I coefficients and traditional performance measures for models derived from the training dataset across imbalance ranging from 1% to 99% of the event class.

New model	Level 3 measure	Training dataset Percentage of the event class						
		1%	10%	30%	50%	70%	90%	99%
**Reference model + ST depression**	**BA**	0.001	0.012	0.034	0.038	0.028	0.008	0.001
	**RB**	**-1.110**	**-0.114**	0.185	0.186	0.087	**-0.138**	**-0.489**
	**I**	0.480	0.505	0.476	0.424	0.382	0.340	0.374
	**LRT p-value**	0.218	0.002	<0.001	<0.001	<0.001	<0.001	0.219
	**BSS**	0.091	0.144	0.193	0.188	0.161	0.100	0.059
	**NRI**	0.877	0.825	0.852	0.848	0.860	0.862	0.884
	**ΔAUC**	0.060	0.082	0.084	0.084	0.085	0.083	0.059
	**ΔF1-score**	0.099	0.264	0.186	0.065	0.017	0.002	0.000
	**ΔMCC**	0.022 (894)	0.222 (371)	0.188	0.172	0.126	0.103 (94)	0.064 (686)
**Reference model + Str Rnd normal**	**BA**	0.001	0.010	0.029	0.036	0.029	0.010	0.001
	**RB**	**-0.731**	**-0.112**	0.133	0.179	0.134	**-0.077**	**-0.821**
	**I**	0.454	0.398	0.394	0.388	0.380	0.376	0.430
	**LRT p-value**	0.209	<0.001	<0.001	<0.001	<0.001	<0.001	0.206
	**BSS**	0.093	0.122	0.167	0.179	0.171	0.133	0.101
	**NRI**	0.926	0.795	0.783	0.775	0.764	0.755	0.855
	**ΔAUC**	0.056	0.079	0.081	0.082	0.081	0.080	0.059
	**ΔF1-score**	0.095	0.216	0.155	0.059	0.032	0.004	0.000
	**ΔMCC**	0.044 (900)	0.199 (372)	0.142	0.137	0.170	0.141 (89)	0.121 (817)
**Reference model** + **Glucose**	**BA**	0.000	0.001	0.001	0.001	0.001	0.001	0.000
	**RB**	**-0.240**	**-0.009**	0.005	0.006	0.004	**-0.010**	**-0.138**
	**I**	0.161	0.119	0.130	0.045	**-0.078**	**-0.152**	**-0.109**
	**LRT p-value**	0.366	0.485	0.437	0.429	0.421	0.425	0.385
	**BSS**	0.023	0.007	0.005	0.006	0.006	0.007	0.014
	**NRI**	0.524	0.150	0.096	0.091	0.106	0.141	0.389
	**ΔAUC**	0.023	0.005	0.003	0.003	0.004	0.007	0.024
	**ΔF1-score**	0.016	0.013	0.002	0.003	0.000	0.000	0.000
	**ΔMCC**	0.026 (916)	0.018 (403)	0.001	0.009	0.002	0.004 (92)	0.015 (846)
**Reference model + Rnd normal**	**BA**	0.000	0.001	0.001	0.001	0.001	0.001	0.000
	**RB**	**-0.260**	**-0.011**	0.006	0.006	0.005	**-0.010**	**-0.190**
	**I**	0.239	0.070	0.059	0.065	0.080	0.096	0.240
	**LRT p-value**	0.456	0.409	0.378	0.384	0.412	0.454	0.487
	**BSS**	0.025	0.009	0.007	0.006	0.007	0.007	0.022
	**NRI**	0.475	0.175	0.140	0.130	0.139	0.164	0.482
	**ΔAUC**	0.023	0.006	0.003	0.003	0.003	0.006	0.020
	**ΔF1-score**	0.016	0.012	0.016	0.000	0.002	**0.000**	0.000
	**ΔMCC**	**-0.018 (917)**	0.017 (384)	0.012	0.005	0.011	0.005 (96)	0.023 (847)

BA coefficients: average absolute changes in prediction between new and reference models; RB coefficients: relative changes in prediction between new and reference models (relative to the reference prediction error); I coefficients: proportions of individuals with prediction changes in each class; LRT, likelihood-ratio test; BSS, Brier skill score; NRI, net reclassification index; ΔAUC, difference in the area under the receiver operating characteristic curves; ΔF1-score, difference in F1-score; ΔMCC, difference in Matthews correlation coefficient.

Shown are mean values from 1000 iterations. Negative values are highlighted in bold type. The number of iterations for which ΔMCC could not be calculated is shown in parentheses.

Results of the LRT were significant for informative variables except at extreme imbalance levels. Across the entire imbalance range, BSS, NRI, ΔAUC and ΔMCC were higher for informative than non-informative variables. ΔF1-score was higher for informative than for non-informative variables, except for imbalance levels of ≥90%, where its values were approximately the same for both informative and non-informative variables. Also, ΔF1-score started to decrease at the imbalance level of 10%.

The weighted overall BA coefficient was higher for informative than non-informative variables across most of the imbalance range, except at extreme imbalance levels. The weighted overall RB coefficient was positive and higher for informative than non-informative variables at balance and intermediate imbalance levels, and it was negative and lower for informative than non-informative variables otherwise. It was negative or fluctuated around zero for non-informative variables. The weighted overall I coefficient was higher for informative than non-informative variables across the entire imbalance range.

ΔMCC was the only measure that could not be calculated for certain iterations at high and extreme imbalance levels, as indicated in parentheses in Table 4. For example, at the imbalance level of 1%, it was calculated for only around 10% iterations.

### Trends in the BA-RB-I coefficients at the three levels of the U-smile method

**Fig 6:**
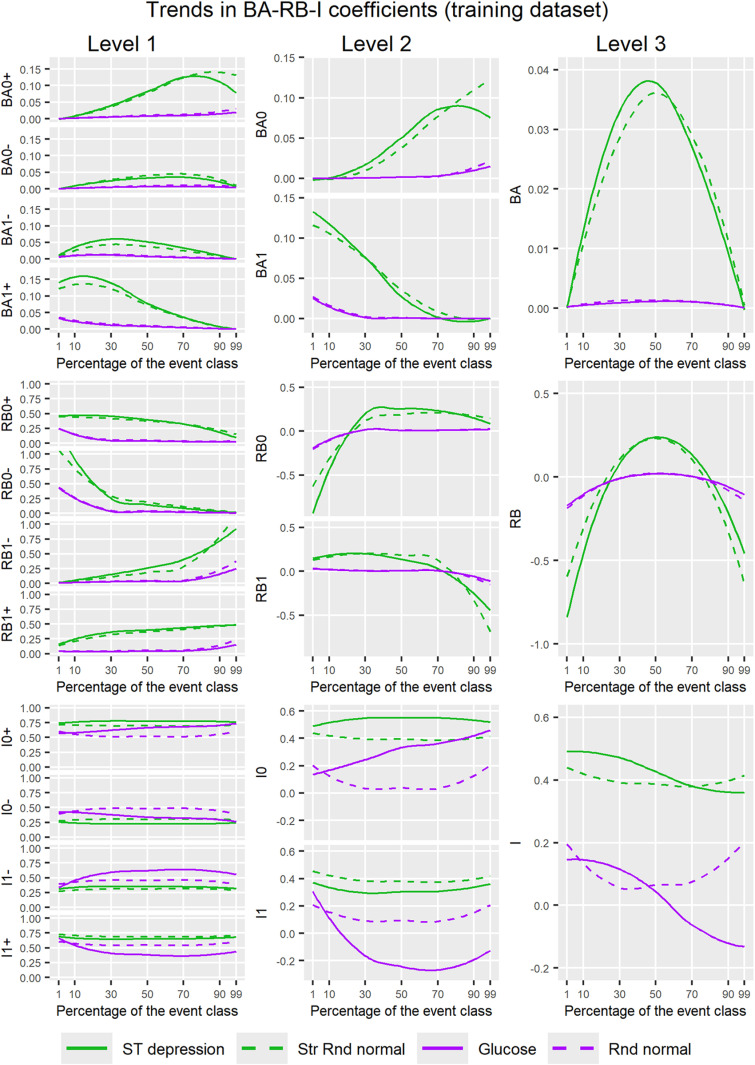
Trends in the BA-RB-I coefficients at the three levels of the U-smile method for four new models derived from the training dataset across imbalance ranging from 1% to 99% of the event class. Two informative variables (ST depression and Str Rnd normal) and two non-informative variables (glucose and Rnd normal) were added to the reference model. Level 1 refers to the subclass-specific coefficients, level 2 to the class-specific net coefficients, and level 3 to the weighted overall coefficients. Smooth curves were fitted using the local polynomial regression fitting (LOESS) method. BA coefficients: average absolute changes in prediction between new and reference models; RB coefficients: relative changes in prediction between new and reference models (relative to the reference prediction error); I coefficients: proportions of individuals with prediction changes in each class.

Figs 6 and S1 show trends in the BA-RB-I coefficients at the three levels of the U-smile method for four new models derived from the training and test datasets, respectively, across imbalance ranging from 1% to 99% of the event class. Trends in the BA-RB-I coefficients demonstrate their ability to distinguish between informative and non-informative variables.

At level 1, the subclass-specific BA and RB coefficients clearly separated informative form non-informative variables across almost the entire imbalance range, except for at extreme imbalance levels, while the I coefficients did so to a small extent. For non-informative variables, the subclass-specific BA coefficients showed no apparent trends and remained approximately constant around zero. For informative variables, $BA_{0}^{+}$ and $BA_{1}^{+}$ (improvement coefficients) increased with decreasing percentages of their respective class in the dataset, while $BA_{0}^{-}$ and $BA_{1}^{-}$ (worsening coefficients) did not show any apparent trends. For informative variables, $RB_{0}^{+}$ and $RB_{1}^{+}$ (improvement coefficients) increased logarithmically with increasing percentages of their respective class in the dataset, while $RB_{0}^{-}$ and $RB_{1}^{-}$ (worsening coefficients) increased exponentially with increasing percentages of their respective class. For non-informative variables, the curves of $RB_{0}^{+}$ and $RB_{0}^{-}$ had similar shapes to those of $RB_{0}^{-}$ for informative variables, but appeared noticeably flatter, as if compressed vertically. The same pattern could be observed for $RB_{1}^{+}$ and $RB_{1}^{-}$ as compared to the curves of $RB_{1}^{-}$ for informative variables. The I coefficients did not show any apparent trends across imbalance and their values differed slightly between informative and non-informative variables. By contrast to the BA-RB coefficients, glucose produced large variability in the I coefficients across imbalance. The trends at level 1 were concordant with the shapes of U-smile (BA) and U-smile (RB) plots.

At level 2, the net BA coefficients clearly separated informative from non-informative variables across almost the entire imbalance range, except for at high and extreme imbalance levels, the net RB coefficients did so across some part of the imbalance range, and the net I coefficients did so across the entire imbalance range (with one exception). For informative variables, the net BA coefficients resembled semi-parabolas (except for BA_0_ for ST depression with the apparent vertex at approximately 80%), increasing as the percentage of their respective class decreased in the dataset. The net BA_0_ and BA_1_ were zero for both informative and non-informative variables at imbalance levels <15% and >80% (values interpolated from the plot), respectively, i.e., when the class they represent was the majority class. Both net RB coefficients followed logarithmic trends for informative variables, increasing as the percentages of their respective class decreased in the dataset. The net RB coefficients intersected their corresponding RB = 0 line at 20–25% for RB_0_ and 75% for RB_1_ (values interpolated from the plot). Sideways to the points of intersection, both net RB coefficients became negative and lower for informative variables than for non-informative variables, indicating prediction worsening. Noteworthy, the net RB_0_ coefficient was approximately constant across the imbalance range from 50% to 99%, and the net RB_1_ coefficient was approximately constant across the imbalance range from 1% to 50%, i.e., when the class they represent was the minority class. This was also reflected in the shapes of U-smile plots, as already described. Both the net BA and RB coefficients showed concordant increasing trends in each class as the percentages of their respective class decreased in the dataset. For non-informative variables, the curves of both the BA and RB coefficients had similar shapes but appeared noticeably flatter, as if compressed vertically compared to the curves of the BA and RB coefficients of informative variables. The net I coefficients did not produce any apparent trends, except for an increasing trend in I_0_ and a parabolic trend in I_1_ for glucose (a non-informative categorical variable). However, the curves of the net I coefficients for informative and non-informative variables moved away from each other compared to level 1, were higher for informative variables and separated informative from non-informative variables better than at level 1. An exception to this was higher I_0_ for glucose than for Str Rnd normal (an informative variable) when the imbalance exceeded approximately 90%.

At level 3, the weighted overall BA coefficients clearly separated informative from non-informative variables across almost the entire imbalance range, except for at extreme imbalance levels, the weighted overall RB coefficients did so across some part of the imbalance range, and the weighted overall I coefficients did so across the entire imbalance range. The weighted overall BA was near zero across the entire imbalance range for non-informative variables. By contrast, for informative variables, the overall BA showed a parabolic shape with a downward opening and the axis of symmetry around 50%. It clearly separated informative from non-informative variables across almost the entire range of imbalance, except for at extreme imbalance levels where the parabolas intersected the BA = 0 line. For both informative and non-informative variables, the weighted overall RB coefficients showed a parabolic shape. However, the parabolas of informative variables were noticeably steeper than those of non-informative variables. Similar to the parabolas of the overall BA coefficient for informative variables, all parabolas of the overall RB coefficients had downward openings and axes of symmetry around 50%. The parabolas of informative variables intersected both the RB = 0 line and the parabolas of non-informative variables at approximately 20–25% and 75–80% of the event class (values interpolated from the plot). Between the intersection points, the overall RB was positive and higher for informative than non-informative variables, while outside this range, it was negative and noticeably lower for informative variables than for non-informative variables, showing the overall worse performance of the new model. Similar to levels 1 and 2, the overall I coefficients did not show any common trends for informative or non-informative variables, but they were noticeably higher for informative variables compared to non-informative variables across the entire imbalance range, and clearly separated informative from non-informative variables.

Results obtained from the test dataset were mostly consistent with those from the training dataset, except for extreme imbalance levels and the RB coefficients, which showed the largest variability between training and test datasets.

## Discussion

We developed the U-smile method, a class-stratified approach, to visualise and evaluate the impact of adding a new variable to a reference model in binary classification (nested models differing by one variable) [16]. The added variable is assessed within the context of the reference set of variables. Consequently, the U-smile method evaluates how the new variable affects the overall model’s predictions. However, it does not differentiate whether the improvement is due to the direct influence of the new variable or its effect on other variables in the model. By comparing the new larger model with the reference one, the U-smile method determines whether the model’s overall performance has improved. If it has, the result is represented by a smiling U-smile plot. The U-smile method can distinguish between informative and non-informative variables, regardless of their type or distribution. Results are presented on U-smile plots of the BA, RB, and I coefficients, which quantify different aspects of comparison between new and reference models, separately for non-events and events. The BA coefficients represent the average absolute prediction changes, the RB coefficients represent relative changes, and the I coefficients represent the proportions of individuals with prediction changes.

In this study, we built on and extended our previous work. We introduced a new level 3, creating a three-level approach of the U-smile method: subclass-specific, class-specific, and overall. The subclass-specific BA-RB-I coefficients of level 1, which are drawn on U-smile plots, and the class-specific net BA-RB-I coefficients of level 2 were introduced in our previous work, where we indicated that the net I_0_ and I_1_ coefficients are equivalent to NRI_0_ and NRI_1_ [16]. In this work, we additionally proposed the weighted overall BA-RB-I coefficients of level 3 that are calculated as weighted means of the corresponding net coefficients with cardinality-based weights to reflect the dataset’s underlying class distribution. We showed that the weighted overall BA coefficient is equivalent to ΔBS. On the other hand, the weighted overall RB and I coefficients are novel but connected to BSS and NRI, respectively, differing by the weights used.

Also, we evaluated the U-smile method across class imbalance of varying levels: extreme imbalance (1% and 99% of the event class), high imbalance (10% and 90%), intermediate imbalance (30% and 70%), and (perfect) balance (50%). The reference model was built using LR with real variables from the Heart Disease dataset, and was compared to four new models that were built by adding: (1) a real and informative variable, (2) a real and non-informative variable, (3) a random and informative variable, and (4) a random and non-informative variable. Random variables were generated from the normal distribution. We compared the U-smile method with certain traditional evaluation measures such as BSS, NRI, ΔF1-score, ΔMCC and ΔAUC of ROC curves.

For each of the three levels, we assessed the sensitivity of the U-smile method to class imbalance by plotting the BA-RB-I coefficients against the percentage of the event class and fitting smooth curves to visualise the trends.

We demonstrated that when used collectively at the three levels of the U-smile method, the BA-RB-I coefficients effectively distinguish between informative and non-informative variables across the entire imbalance range. Also, the RB coefficients indicate the reduction of the reference model’s overfitting to the majority class (in this study, it was observed when the percentage of the majority class in the dataset was approximately 75% and more).

### Insights from the three-level approach of the U-smile method

The U-smile method compares new and reference models at three levels of generality, providing separate BA, RB and I coefficients for four subclasses (level 1), two classes (level 2) and the overall dataset (level 3). The BA coefficients represent average absolute changes in prediction between new and reference models, the RB coefficients – relative changes in prediction between new and reference models (relative to the reference prediction error), and the I coefficients – the proportions of individuals with prediction changes in each class. The insights derived from the U-smile method’s coefficients vary across these levels. In this study, we analysed the effect of class imbalance across all three levels. To facilitate interpretation and generalisation, we used trend plots of the BA-RB-I coefficients for each level.

Level 1 provides the most detailed analysis. The effect of adding a new variable to the reference model is quantified separately for non-events and events, further split into subclasses with improved and worsened prediction. This is visualised on the U-smile (BA) and U-smile (RB) plots, with the I coefficients as a weighing factor for point size (Figs 4 and 5). No other known method for comparing binary prediction models provides such unique information for these four subclasses.

Level 2 provides an intermediate degree of generality. The U-smile method measures the net prediction changes between the new and reference models, and evaluates non-events and events separately. This approach is similar to that used in NRI_0_ and NRI_1_ [19,31], and stratified BS [25], all of which also evaluate non-events and events separately. Although the stratified BS was originally introduced for evaluating a single model, and not for comparing two models, it is clear that the net BA coefficients are equivalent to the difference in stratified BS in each class.

Level 3 provides the most general evaluation. The overall effect of a new variable is evaluated with single weighted BA, RB and I coefficients. This approach is analogous to using single measures such as ΔBS, BSS, NRI, ΔF1-score, ΔMCC and ΔAUC of ROC curves. This level of analysis provides a comprehensive summary if adding a new variable to the reference model has globally improved or worsened its performance. The weighted overall BA-RB-I coefficients are calculated as weighted means of their respective net coefficients using cardinality-based weights. As a result, the weighted overall BA is equivalent to ΔBS (as shown in our previous work), and the weighted overall RB and I are related to BSS (for which residual error weights are used with the net RB) [16] and NRI (which is unweighted), respectively. The weighted overall I coefficient is, therefore, a weighted version of the unweighted NRI.

In this study, we compared the weighted overall BA-RB-I coefficients with unweighted measures. Unweighted measures assign equal importance to both classes regardless of the dataset’s class distribution and artificially inflate the contribution of the minority class. By contrast, we defined the weighted overall BA-RB-I coefficients using cardinality-based weights to ensure that the underlying class distribution is adequately represented. In the minority class, as shown by the net BA, net RB and net I coefficients in this class, it was noticeably easier to distinguish informative from non-informative variables, and this was achieved with high precision based solely on these net coefficients. Artificial inflation of the minority class’s influence that is not reflected in the dataset’s class distribution might have been the reason why the unweighted BSS separated the informative from non-informative variables better than the weighted overall BA-RB coefficients. Therefore, researchers using overall coefficients should be aware of the influence of individual components (i.e., the majority and minority classes) on the obtained result and not use unweighted coefficients by default, but only as a result of a conscious decision to treat both classes equally. When used collectively at the three levels, the BA, RB and I coefficients effectively differentiate between informative and non-informative variables across the entire imbalance range. The I coefficients showed an increasing ability to separate informative from non-informative variables across the three levels and may find their best use at level 3. For informative variables and at higher imbalance levels, the net BA coefficients clearly showed prediction improvement in the minority class, while the net RB coefficients clearly showed prediction worsening in the majority class. Therefore, we can say that the net BA is worth using when we focus on the minority class and the net RB – on the majority class.

The net and weighted overall RB coefficients were the only coefficients with noticeably negative values. The net RB coefficients measure net relative changes in prediction between new and reference models, compared to the prediction error of the reference model in each class. While their negative values indicate a worse prediction (larger prediction error) of the new model, they must be interpreted in the context of the reference model. Namely, the reference model was overfitted to the majority class at higher imbalance levels, i.e., the predicted probabilities were extremely close to the true outcome. This was indicated by extremely low, near-zero values of the stratified BS in the majority class, very high values of the stratified BS in the minority class, and large differences between test and training datasets in the minority class (Fig 3 and Table 2). Therefore, a worse prediction of the new model, as indicated by negative RB coefficients, can be interpreted as a reduction in the reference model’s overfitting. Interpolating from trends plots (Fig 6), we observe that adding a new variable to the reference model reduced overfitting when the percentage of the majority class in the dataset was approximately 75% and more.

Although the net BA coefficients showed improvement in the minority class, the negative values of the net RB coefficient in the majority class might have been the reason why the LRT was insignificant for informative variables at extreme imbalance levels. This was due to a different approach to model comparison of the RB coefficients and LRT. The LRT compares the goodness of fit of two models, i.e., how close the predicted probabilities are to the true outcome. As the overfitting to the majority class was reduced, the fit in the majority class was worsened, i.e., the predicted probabilities moved further from the true outcome in the new model. Although adding informative variables was insignificant according to the LRT, the reference model was improved qualitatively (the I coefficients) in both classes and also quantitatively (the BA and RB coefficients) in the minority class. All this may have a beneficial effect in terms of model generalizability and potential for practical use, which is especially important in real-life problems.

### The U-smile method vs. ΔBS, BSS, NRI, ΔF1-score and ΔMCC

There are different types of metrics for comparing prediction models, including but not limited to counting measures (e.g., sensitivity, specificity, NRI, ΔF1-score, ΔMCC, I coefficients), quantitative measures (ΔBS, BSS, BA and RB coefficients) and ranking measures (e.g., ROC curve). Counting measures focus on the frequencies and can be based on a confusion matrix with a preselected classification threshold (ΔF1-score, ΔMCC) or number of prediction changes (NRI, I). Quantitative measures are based on distances between the true class and predicted probability. Each type of metric has its advantages and limitations. This study compared the U-smile method with BSS, NRI, ΔF1-score and ΔMCC. Because of the suboptimal performance of sensitivity and specificity in highly imbalanced datasets (as observed in preliminary analysis, results not shown), these measures were excluded from this study.

While counting measures are often more interpretable than other metrics and relatively easy to calculate, their main limitations include sensitivity to class imbalance, susceptibility to noise and providing limited information (as they omit the magnitude of errors or distribution of predictions). Although quantitative measures can be sensitive to outliers as they focus on average errors and difficult to interpret, they consider subtle changes in model predictions and allow for a more accurate comparison of models. However, most traditional measures are not class-stratified, i.e., they assess models as a whole, missing information on each class.

To address these limitations, it is recommended to use a combination of quantitative and counting measures [32]. The U-smile method provides a unique approach to model comparison, with both quantitative (BA and RB) and counting (I) measures for each class. This combined approach enables a comprehensive comparison of models, considering both prediction accuracy (model residuals) and the relative frequency of prediction changes. Another advantage is a straightforward and intuitive interpretation of the U-smile plot, with smiles showing improvement and frowns – worsening in prediction.

Unlike confusion matrix-based metrics (e.g., F1-score, MCC, sensitivity, specificity), which require a predefined classification threshold (conventionally of 0.5 [27], which is considered unsuitable for high imbalance levels [27]), the U-smile method is threshold-independent. It is a significant advantage in comparative studies where inconsistent thresholding hinders comparing the results as it eliminates inconsistencies resulting from varying threshold choices.

Although MCC requires classification threshold, it is often considered the most suitable confusion matrix-based measure for moderately imbalanced datasets and is frequently used as an alternative to the ROC curve [21,33,34]. In fact, the U.S. Food and Drug Administration (FDA) recommends using MCC as the primary evaluation measure in MicroArray Quality Control (MAQC) and Sequencing Quality Control (SEQC) projects [35,36]. A claimed advantage of MCC is that it uses values from all four quadrants of the confusion matrix, i.e., TP, FN, FP and TN. Analogously, the U-smile method involves four subclasses: events with better prediction are conceptually analogous to TP, events with worse prediction to FN, non-events with worse prediction to FP, and non-events with better prediction to FN. However, the U-smile method’s coefficients are calculated differently than the confusion matrix-based measures.

Unlike MCC, the F1-score uses values from only three quadrants of the confusion matrix, i.e., TP, FN and FP, and neglects important information of TN. As a result, it is sensitive to class swapping, i.e., when the event class is relabelled non-event and vice versa, while MCC is unaffected by class swapping [37]. F1-score is invariant to class swapping only when TP = TN. In fields with extremely large numbers of TN, like DNA sequence analysis [38], the F1-score’s omission of TN, though sometimes justifiable, hinders a comprehensive model assessment. Our study also highlighted the limitations of the F1-score. Namely, the F1-score of the reference model increased across the entire imbalance range, approaching its theoretical maximum at 99% of the event class. This resulted in a decreasing trend of ΔF1-score for informative variables, starting from 10% of the event class.

By contrast, the BA, RB and I coefficients of levels 1 and 2 provide separate assessments in each class, thus avoiding the issue of class swapping entirely. Moreover, the overall BA, RB and I coefficients of level 3 are invariant to class swapping. In this way, they preserve all information, which is particularly advantageous for imbalanced data.

Confusion matrix-based metrics can become unreliable or even incalculable in highly imbalanced data. In this study, we frequently encountered this issue in the case of ΔMCC. At high and extreme imbalance levels, it was not calculated for the average of over 60% (range 9% to 93%) of iterations due to zero values in the denominator. This raises significant concerns about reliability of ΔMCC under severe class imbalance. By contrast, the U-smile method’s threshold-free BA-RB-I coefficients are always calculable, regardless of class imbalance, as long as the new and reference models return different predictions. In this study, the RB coefficients of levels 1 and 2 were not calculated for the minority class only in 3 out of 8000 iterations (0.0375%). This was the case for glucose at an imbalance of 99% and on the test dataset. Therefore, we recommend caution when evaluating classification based simply on TP, FN, FP and TN.

### The U-smile method vs. ROC curve

The ROC curve, a ranking measure, is considered a measure of discrimination between classes [39]. It is created by plotting the true positive rate (sensitivity) on the y-axis and the false positive rate (1-specificity) on the x-axis, determined for varying classification thresholds based on model predictions. Although the AUC of a ROC curve is generally easy to interpret, the concept behind the ROC curves can be more difficult to understand. Also, the ROC curve evaluates the overall model, as by definition it is not stratified by class. While plotting two separate ROC curves of the new and reference models in a single plot remains the most commonly used graphical method for model selection [21,40], it can be difficult to visually compare a series of ROC pairs plots, especially when their AUCs are similar. As already shown, the U-smile plot offers a more intuitive interpretation than the plot of ROC curves in model comparison.

Although the PR curve offers an alternative to the ROC curve in imbalanced datasets, we chose not to include it in this study. Similar to the F1-score, it focuses primarily on the event class (TN from the confusion matrix are not used to build the PR curve), thus leading to an incomplete model evaluation and being sensitive to class swapping.

The ROC curve has been widely criticised for its poor performance on imbalanced data. In particular, the ROC curve can overestimate the classifier’s performance at higher imbalance levels. In this study, we observed overly optimistic values of AUC at extreme imbalance levels (S2 and S3 Figs). For informative variables, ΔAUC remained approximately constant (ranging from 0.079 to 0.085) across the imbalance range from 10% to 90% (Table 4), and the corresponding plots of ROC curves were quite similar (S2 and S3 Figs). By contrast, the U-smile plots produced different shapes for each imbalance level and provided separate assessments in each class, thus providing more insight into the effect of class imbalance.

The advantage of the U-smile method over the ROC curve makes it a particularly suitable method in imbalanced classification. In many real-life problems of imbalanced classification, it is often insufficient to determine that a new variable has improved the overall performance of the reference model. For example, in rare diseases or fraud detection, the minority class (events) is of primary interest, while in screening tests, the majority class (non-events) is of interest. Showing how each class is affected by the new variable, determining the magnitude of prediction changes and the number of individuals with changed predictions is particularly important. The U-smile plot summarises these results and enables a swift visual assessment. Therefore, the U-smile method overcomes the ROC curve’s limitations, especially in imbalanced datasets.

### The U-smile method vs. SHAP

In addition to the selected traditional methods and metrics for variable selection (ROC curve, ΔBS, BSS, NRI, ΔF1-score and ΔMCC), we evaluated the U-smile method by comparing it with SHapley Additive exPlanations (SHAP), a recent approach to explaining model predictions [41,42]. SHAP provides local explanations of how each variable contributes to individual predictions by distributing the prediction difference from a baseline model among the input variables based on principles from cooperative game theory. SHAP proved effective in interpreting predictive models in the medical context to identify highly contributing predictors of acute kidney injury [43], procedure-related mortality and unplanned readmission [44], for example. S4 Table shows a comparison of methods for variable selection in terms of interpretability, effectiveness in imbalanced datasets, computational complexity, dependence on classification threshold, dedicated applicability and origin and graphical representation.

To compare the U-smile method with SHAP, we plotted the SHAP values for four new models derived from the training dataset across imbalance ranging from 1% to 99% (S4 Fig). Noteworthy, the SHAP values alone do not reliably distinguish between informative (ST depression and Str Rnd normal) and non-informative variables (glucose and Rnd normal) at extreme imbalance levels (1% and 99%). This is likely due to the limited number of cases in the minority class, which reduces the variability of SHAP values and makes it challenging to discern meaningful patterns. By contrast, the U-smile plots presented clear differences between informative and non-informative variables even at extreme imbalance levels.

Moreover, at high and intermediate imbalance levels (10% and 30% of the event class), the SHAP plots indicate that adding an informative variable generally increases the predicted risk for the minority class, consistent with the results of the U-smile method. In particular, the SHAP values suggest that the new variable contributes more strongly to individual predictions in the minority class, reflecting a greater overall improvement in prediction for this group. This aligns with the class-specific analysis provided by U-smile, which separately highlights the improvement (or worsening) in prediction for non-events and events.

While SHAP offers valuable, fine-grained insights into how individual features influence predictions, it may not be as effective as the U-smile method in detecting subtle improvements under extreme class imbalance. By contrast, the U-smile method’s class-stratified perspective and focus on changes from a reference model provide a more robust approach for evaluating model performance in highly imbalanced datasets. Both the U-smile method and SHAP provide an intuitive graphical representation of results. The U-smile method facilitates a more nuanced understanding of a predictor’s effects by identifying four distinct subclasses: non-events that will benefit from the predictor, non-events that will not, events that will benefit, and events that will not. This ability to explain the nuanced impact of a predictor on different outcomes highlights the U-smile method’s potential for contributing to the emerging field of explainable ML (XML) and explainable artificial intelligence (XAI). However, further dedicated investigation is required to fully explore this potential.

### Study limitations

We evaluated the U-smile method across seven pre-defined imbalance levels of 1%, 10%, 30%, 50%, 70%, 90% and 99% of the event class. While results for intermediate levels can be estimated through interpolation, only results for the seven levels are presented in tables due to space limitations. Based on preliminary analysis (results not shown), we decided to compare the U-smile method with some standard measures (BSS, NRI, ΔAUC, ΔF1-score, ΔMCC), and to exclude others (e.g., sensitivity and specificity) from this study.

Unlike confusion matrix-based metrics, the U-smile method does not require specifying a classification threshold as it is based directly on the model’s probability estimates. As a result, it can only be applied to ML algorithms that generate probability estimates (e.g., LR, random forest, neural networks with softmax activation function) and those that require some transformation to do so (decision trees).

To maintain consistency with our previous work and due to space limitations, we evaluated the U-smile method using only LR models in this study. LR is biased towards the majority class and sensitive to class imbalance [45], which was visible in the stratified BS of the reference model and immediately reflected in the shapes of the U-smile (BA) and U-smile (RB) plots. Also, we only compared nested models that differed by exactly one variable (nested setting).

Although we have not explicitly assessed the computational complexity of the U-smile method or the hardware aspects, neither significant computational resources nor the amount of time was required for computations in this study. However, if the U-smile method is used with machine learning algorithms that are more computationally complex than LR or may require preprocessing steps or additional regularisation techniques to improve the model’s robustness [46–49], e.g., neural networks with large datasets or high-resolution images, the training time can be significantly longer. Then, the final execution time will mostly depend on the machine learning algorithm used.

Also, we have not applied the U-smile method to problems where the data are generated in real-time, and the outcome must be constantly and efficiently recalculated. A notable application is in monitoring vital signals, such as ECG data in high-risk patients to detect life-threatening arrhythmias or atrial fibrillation. It could also be used for real-time tracking of athletes, either on the track or field, to identify early signs of exhaustion and prevent sports-related injuries. Additional potential applications include analysing sleep data, heart rate and heart rate variability, cortisol and glucose levels, and body temperature. Integrating the U-smile method in these contexts could enable faster, more effective visualization of dynamic changes in binary outcomes, thereby enhancing predictive accuracy and supporting better decision-making. Another application is in computer vision and autonomous vehicles, where parallel computation and specialized hardware implementations may be required, especially for users with limited hardware resources [50–57].

We evaluated the U-smile method on training and test datasets, comprising 300 and 100 observations, respectively. We did not investigate the impact of dataset size on values and variability of the BA-RB-I coefficients. Similar to other model evaluation metrics, smaller dataset sizes would most likely result in higher variability of coefficients and less stable results.

We deliberately used non-informative and highly informative variables, and we are aware that the results of this study regarding the ability of the U-smile method to distinguish between informative and non-informative variables may be optimistic. Using informative variables of less predictive strength may reduce the magnitude of the observed differences. However, the artificial random variables generated comparable results to the real variables from the Heart Disease dataset.

Although negative values of the net RB coefficients express the reduction of the reference model’s overfitting to the majority class, the U-smile method does not assess the potential remaining overfitting of the new model as it evaluates the differences between new and reference models.

### Perspectives

There are many possible directions and challenges for future investigations to understand the U-smile method further and expand its use:

- Applying the U-smile method to other datasets across different domains beyond clinical setting to assess its generalizability;
- Comparing the U-smile method with other model comparison methods on the same dataset to identify more of its strengths and weaknesses;
- Applying the U-smile method to ML algorithms other than LR on the same dataset as other ML algorithms may be more robust to class imbalance;
- Investigating the effect of dataset size on the BA-RB-I coefficients;
- Applying the U-smile method to compare nested models that differ by more than one new variable;
- Applying the U-smile method for stepwise regression. The U-smile plots can be shown as small images, e.g., postage stamp size. The best candidate variables can be instantly identified with a quick visual examination of a series of U-smile plots. Until now, there has been a lack of similar methods that provide a graphical and concise summary of the variable selection process;
- Assessment of the computational complexity of the U-smile method;
- Applying the U-smile method to choose hyperparameters of neural networks, such as the number of channels, attention parameters and compression rates.

The application of the U-smile method to ML algorithms beyond LR may illustrate robustness to class imbalance. Moreover, the integration of the U-smile method with LR or other ML algorithms, such as random forests or neural networks with soft-max activation functions, introduces new features that improve interpretability. These methods simplify and present results more intuitively. While all these approaches assist in predictor selection and summarizing predictive value, the U-smile method uniquely visualizes the effects of adding such a parameter on specific subclasses, providing information at various levels of detail.

## Conclusions

The U-smile method, with its U-smile plot and BA-RB-I coefficients, offers a three-level approach to evaluating the effect of a new variable added to the predictive model for binary classification, i.e., the subclass-specific level 1, class-specific level 2, and overall level 3. By combining both graphical assessment (the U-smile plot) and numerical assessment (quantitative measures, i.e., the BA and RB coefficients, and counting measures, i.e., the I coefficients), the U-smile method enables a comprehensive comparison of new and reference models. It distinguishes between informative and non-informative variables across class imbalance ranging from 1% to 99% of the event class in the dataset. At higher imbalance levels, the net RB coefficients can additionally express reduced overfitting to the majority class.

The U-smile method allows for a swift visual assessment of multiple new variables in each class across imbalance. The U-smile plots of the subclass-specific BA-RB-I coefficients are straightforward and intuitive to interpret, with smiles indicating prediction improvement and frowns – prediction worsening. A joint analysis of the U-smile (BA) and U-smile (RB) plots’ shapes enables a detailed analysis of prediction changes in each class, making it extremely easy to identify prediction improvement in the minority class, and reduction of the reference model’s overfitting to the majority class.

Unlike many traditional evaluation metrics, the U-smile method is classification threshold-independent and stratified by subclass and class at levels 1 and 2, respectively, providing a deeper insight into variable selection. Cardinality-based weights for the overall BA-RB-I coefficients of level 3 accurately reflect the underlying class distribution in the dataset. The U-smile method has the potential to outperform the LRT in correctly identifying informative variables at extreme imbalance levels.

The U-smile method proved highly effective in variable selection for imbalanced binary classification, making it a useful tool for real-life problems, where imbalanced datasets are prevalent.

## Supporting information

S1 Table Level 1: Values of the subclass-specific BA-RB-I coefficients for new models derived from the training and test datasets across imbalance ranging from 1% to 99% of the event class. (XLSX)

S2 Table Level 2: Values of the class-specific net BA-RB-I coefficients for models derived from the test dataset across imbalance ranging from 1% to 99% of the event class. (XLSX)

S3 Table Level 3: Values of the weighted overall BA-RB-I coefficients and traditional performance measures for models derived from the test dataset across imbalance ranging from 1% to 99% of the event class. (XLSX)

S4 Table A comparison of methods for variable selection. Criteria such as interpretability, effectiveness in imbalanced datasets, computational complexity, dependence on classification threshold, dedicated applicability and origin and graphical representation were used for the comparison.
(XLSX)

S1 Fig Trends in the BA-RB-I coefficients at the three levels of the U-smile method for four new models derived from the test dataset across imbalance ranging from 1% to 99% of the event class. Two informative variables (ST depression and Str Rnd normal) and two non-informative variables (glucose and Rnd normal) were added to the reference model. Level 1 refers to the subclass-specific coefficients, level 2 to the class-specific net coefficients, and level 3 to the weighted overall coefficients. Smooth curves were fitted using the local polynomial regression fitting (LOESS) method. BA coefficients: average absolute changes in prediction between new and reference models; RB coefficients: relative changes in prediction between new and reference models (relative to the reference prediction error); I coefficients: proportions of individuals with prediction changes in each class.
(TIFF)

S2 Fig The ROC curves of the reference and new models derived from the training dataset across imbalance ranging from 1% to 99% of the event class. For each imbalance level, the mean Brier score of the reference model was calculated from 1000 iterations. The ROC curves were then plotted for the iteration in which the reference Brier score was closest to the mean value.
(TIFF)

S3 Fig The ROC curves of the reference and new models derived from the test dataset across imbalance ranging from 1% to 99% of the event class. The ROC curves were plotted using the same iterations as those used for the training dataset.
(TIFF)

S4 Fig The SHAP values for four new models derived from the training dataset across imbalance ranging from 1% to 99% of the event class. Two informative variables (ST depression and Str Rnd normal) and two non-informative variables (glucose and Rnd normal) were added to the reference model. The SHAP values show the strength and direction of the influence of a given variable on the prediction: Positive values (>0) mean that the added variable increases the probability of the event class, negative values (<0) mean that the added variable decreases the probability of the event class. The greater the absolute value, the stronger the influence of the new variable.
(TIF)
