# Supplementary figures and images for: Evaluating the three-level approach of the U-smile method for imbalanced binary classification
Source: PLoS One. 2025 Apr 10;20(4):e0321661. doi: 10.1371/journal.pone.0321661 (PMC11984743; doi:10.1371/journal.pone.0321661)

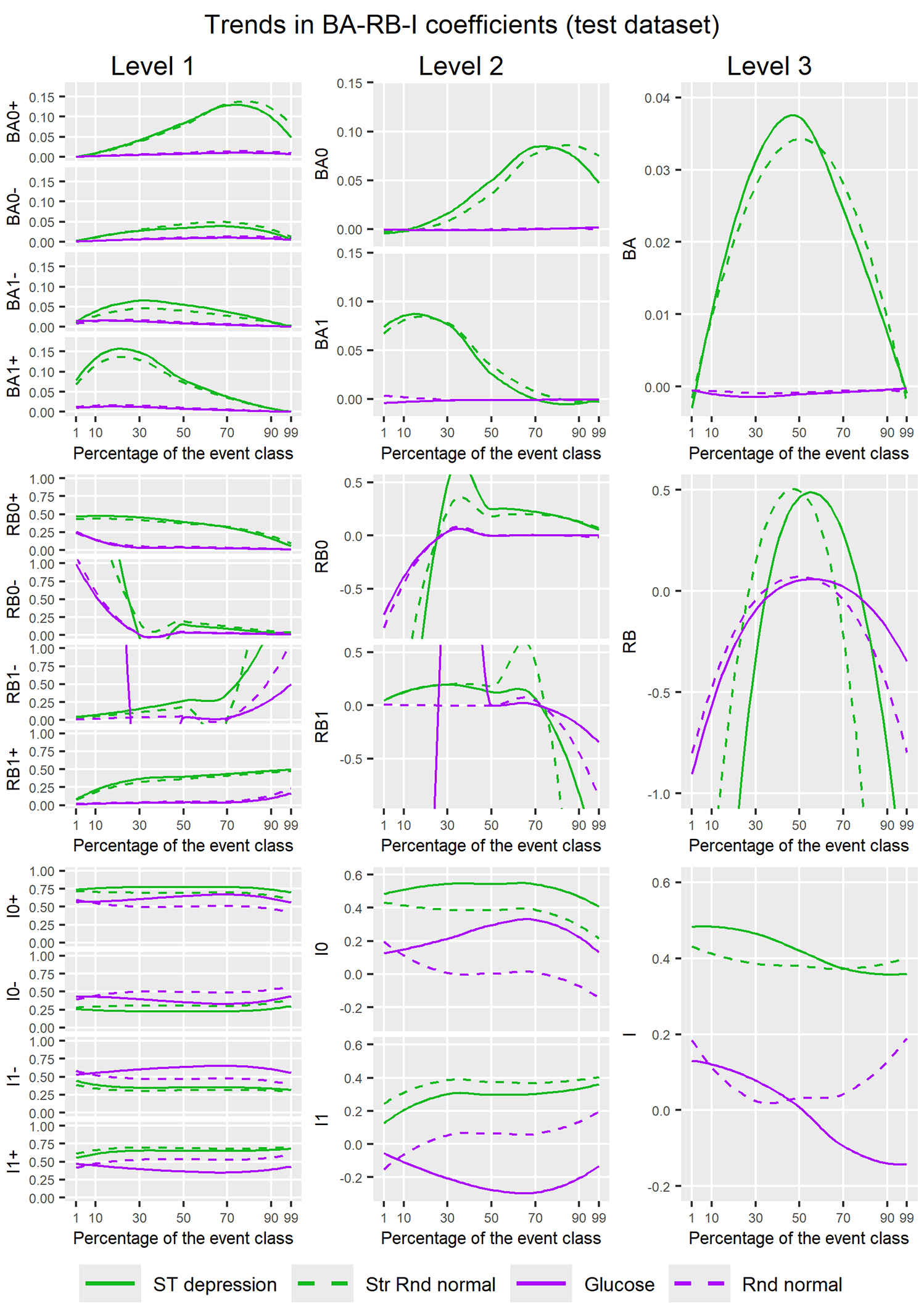

Supplement: S1 Fig — Two informative variables (ST depression and Str Rnd normal) and two non-informative variables (glucose and Rnd normal) were added to the reference model. Level 1 refers to the subclass-specific coefficients, level 2 to the class-specific net coefficients, and level 3 to the weighted overall coefficients. Smooth curves were fitted using the local polynomial regression fitting (LOESS) method. BA coefficients: average absolute changes in prediction between new and reference models; RB coefficients: relative changes in prediction between new and reference models (relative to the reference prediction error); I coefficients: proportions of individuals with prediction changes in each class. (TIFF) [file pone.0321661.s005.tiff]

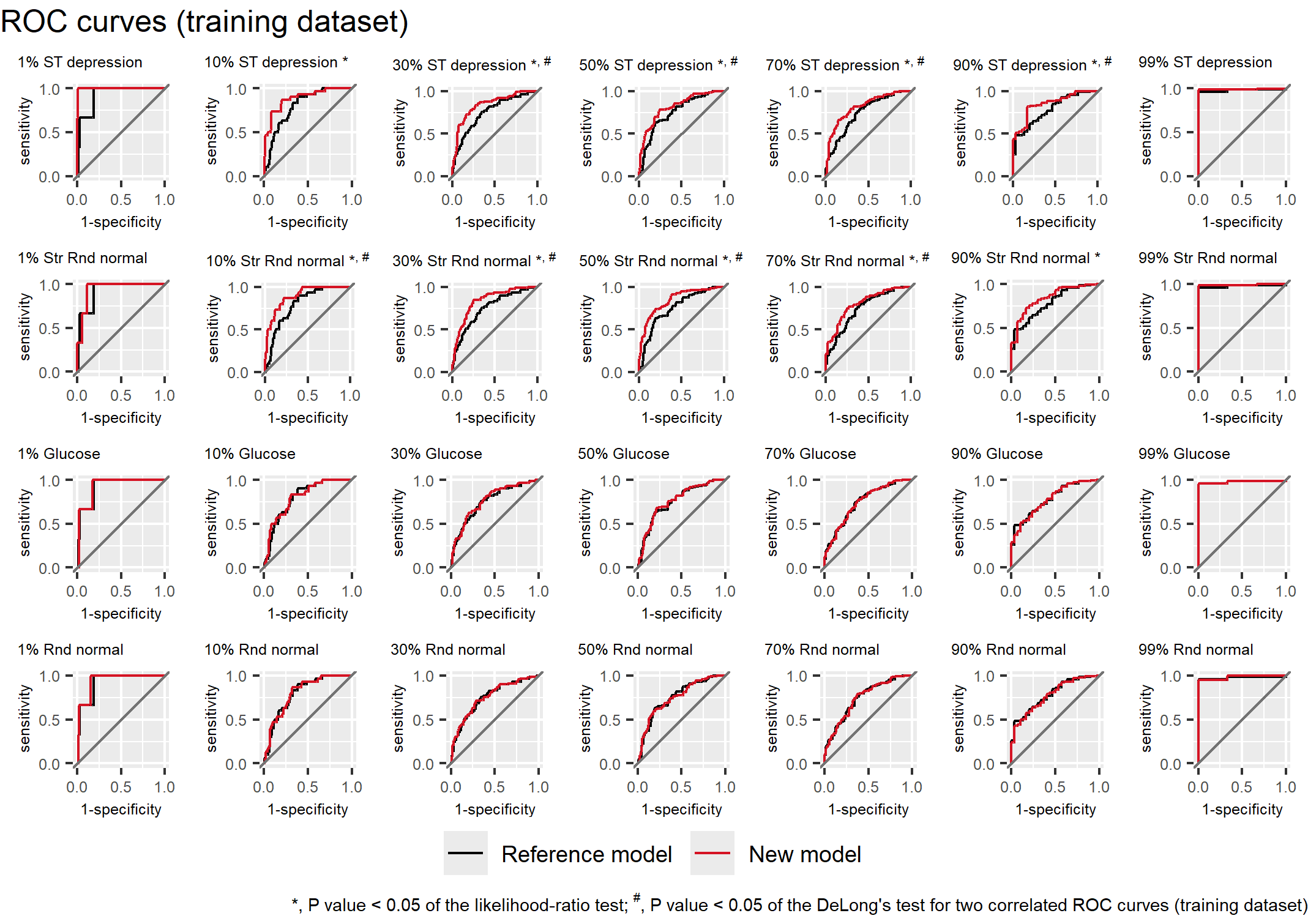

Supplement: S2 Fig — For each imbalance level, the mean Brier score of the reference model was calculated from 1000 iterations. The ROC curves were then plotted for the iteration in which the reference Brier score was closest to the mean value. (TIFF) [file pone.0321661.s006.tiff]

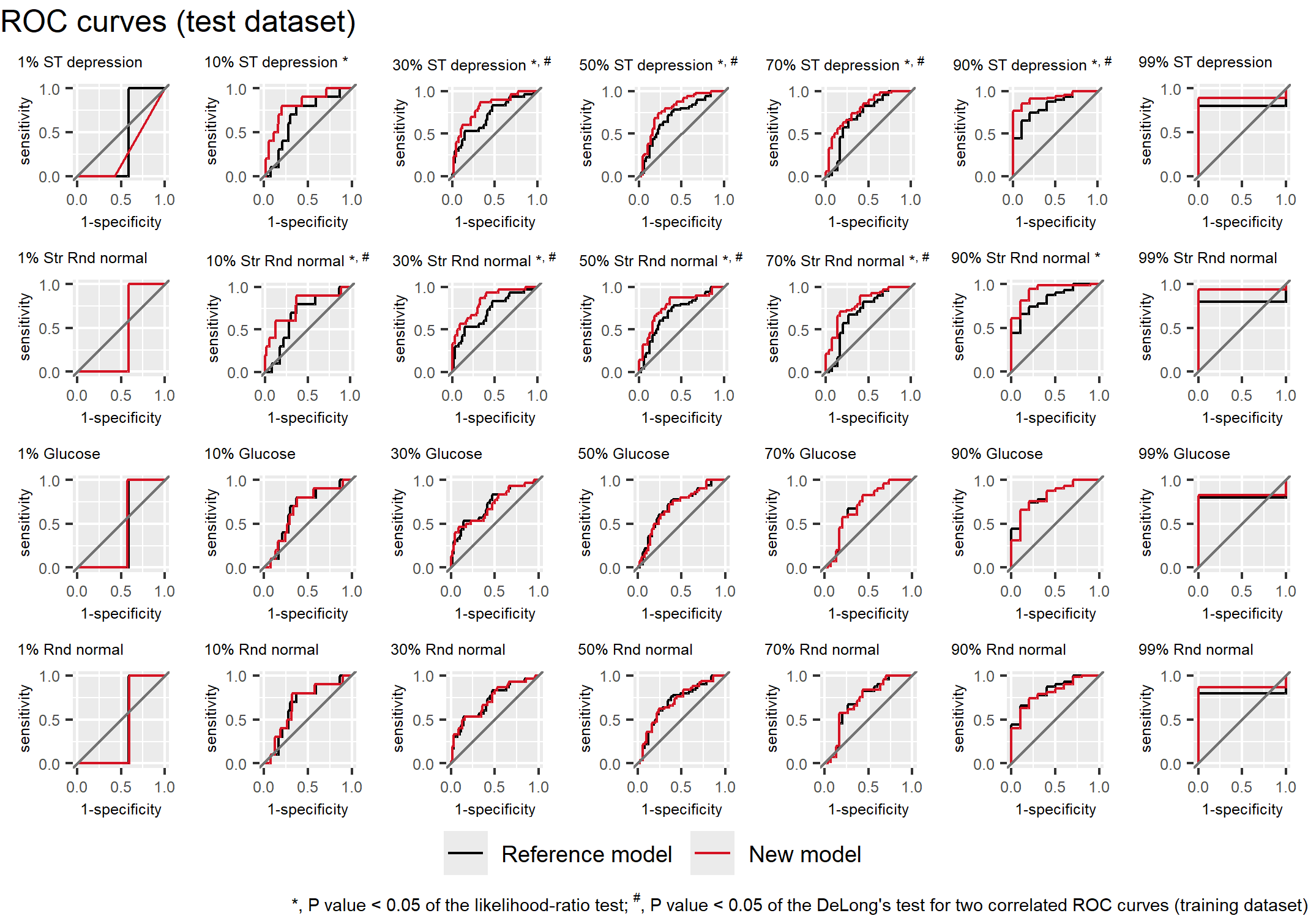

Supplement: S3 Fig — The ROC curves were plotted using the same iterations as those used for the training dataset. (TIFF) [file pone.0321661.s007.tiff]

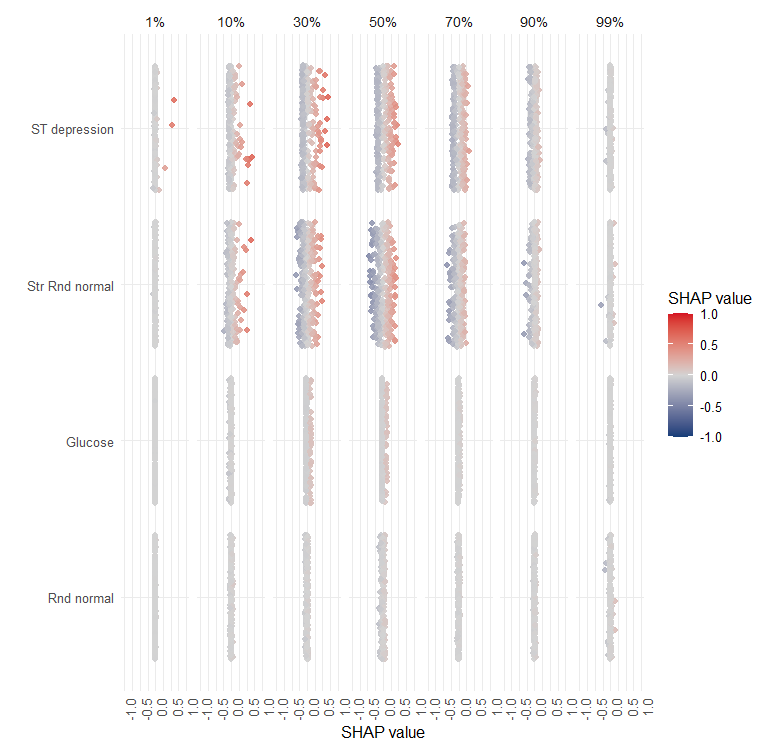

Supplement: S4 Fig — Two informative variables (ST depression and Str Rnd normal) and two non-informative variables (glucose and Rnd normal) were added to the reference model. The SHAP values show the strength and direction of the influence of a given variable on the prediction: Positive values (>0) mean that the added variable increases the probability of the event class, negative values (<0) mean that the added variable decreases the probability of the event class. The greater the absolute value, the stronger the influence of the new variable. (TIF) [file pone.0321661.s008.tif]
